# Fluid excess on intensive care unit after mechanical thrombectomy after acute ischemic stroke is associated with unfavorable neurological and functional outcomes: An observational cohort study

**DOI:** 10.1177/23969873241271642

**Published:** 2024-08-16

**Authors:** Maximilian Schell, Christina Mayer, Marcel Seungsu Woo, Hannes Leischner, Marlene Fischer, Jörn Grensemann, Stefan Kluge, Patrick Czorlich, Christian Gerloff, Jens Fiehler, Götz Thomalla, Fabian Flottmann, Nils Schweingruber

**Affiliations:** 1 Department of Neurology, University Medical Center Hamburg-Eppendorf, Hamburg, Germany; 2 Institute of Neuroimmunology and Multiple Sclerosis (INIMS), Center for Molecular Neurobiology Hamburg (ZMNH), University Medical Center Hamburg-Eppendorf, Hamburg, Germany; 3 Department of Neuroradiology, University Medical Center Hamburg-Eppendorf, Hamburg, Germany; 4 Department of Intensive Care Medicine, University Medical Center Hamburg-Eppendorf, Hamburg, Germany; 5 Department of Neurosurgery, University Medical Center Hamburg-Eppendorf, Hamburg, Germany

**Keywords:** Stroke, thrombectomy, fluid management, fluid balance, intensive care unit

## Abstract

**Introduction:**

Endovascular thrombectomy stands as a pivotal component in the standard care for patients experiencing acute ischemic stroke with large vessel occlusion. Subsequent care for patients often extends to a neurological intensive care unit. While fluid management is integral to intensive care, the association between early fluid balance and neurological and functional outcomes post-thrombectomy has not yet been thoroughly investigated.

**Methods:**

In a retrospective analysis of an observational, single-center study spanning from 2015 to 2021 at the University Medical Center Hamburg-Eppendorf, Germany, we enrolled stroke patients who underwent thrombectomy and received subsequent treatment in the ICU. Unfavorable functional and neurological outcome was defined as a mRS > 2 on day 90 after admission (mRS d90) or NIHSS > 5 at discharge, respectively. A multivariate regression model, adjusting for confounders, utilized the average fluid balance in the first 5 days to predict outcomes. Patients were dichotomized by their average fluid balance (>1 L vs <1 L) within the first 5 days, and a multivariate mRS d90 shift analysis was conducted after adjusting for covariates.

**Results:**

Between 2015 and 2021, 1252 patients underwent thrombectomy, and 553 patients met the inclusion criteria (299 women [54%]). Unfavorable functional outcome was significantly associated with a higher daily average fluid balance in the first 5 days in the ICU (mRS d90 ⩽ 2: 0.3 ± 0.5 L, mRS d90 > 2: 0.7 ± 0.7 L, *p* = 0.02). The same association was observed for the NIHSS at discharge (NIHSS ⩽ 5: 0.3 ± 0.5 L; NIHSS > 5: 0.6 ± 0.6 L; *p* = 0.03). The mRS d90 shift analysis revealed significance for patients with an average fluid balance <1 L for better functional outcomes (adjusted odds ratio [AOR] 2.17; 95% confidence interval [CI] 1.54–3.07; *p* < 0.01).

**Discussion:**

Fluid retention in post-thrombectomy stroke patients in the ICU is associated with poorer functional and neurological outcomes. Consequently, fluid retention emerges as an additional potential predictor for post-intervention stroke outcomes. Our findings provide an initial indication that preventing excessive fluid retention in stroke patients after endovascular thrombectomy could be beneficial for both functional and neurological recovery. Therefore, fluid retention might be an element to consider in optimizing fluid management for stroke patients.

## Graphical abstract

**Graphical abstract:**
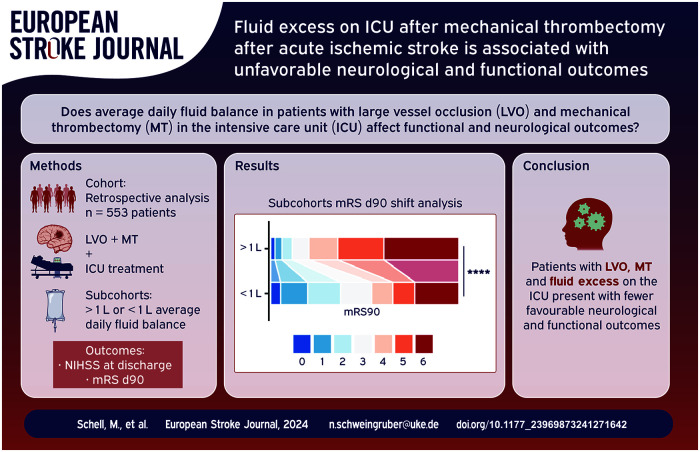

## Introduction

The landscape of acute ischemic stroke treatment has been revolutionized by image-guided intravenous thrombolysis therapy and the evolution of mechanical endovascular interventions.^1^ Over the recent years, the scope of endovascular thrombectomy has expanded, enabling the treatment of large vessel occlusions in both the anterior and posterior circulation.^2,3^ In the near future, further advances including the treatment of medium vessel occlusions and larger infarct core volumes are expected to broaden the indications for endovascular thrombectomy. This evolution is likely to result in an increased use of endovascular treatments in clinical practice.^4,5^ Concurrently, given that ischemic stroke predominantly affects the elderly, comorbidities such as hypertension, heart failure, diabetes mellitus, kidney disease, and cancer are frequently present in patients undergoing mechanical thrombectomy.^6–8^ Particularly in older and less compliant patients experiencing longer and more complex interventions, general anesthesia, rather than conscious sedation, followed by mechanical ventilation is necessary.^9^ Consequently, patients often need post-interventional transfer to an intensive care unit (ICU) to continue mechanical ventilation, and in some instances, to address additional complications such as hemodynamic instability or infections.

A crucial aspect of comprehensive patient care in the ICU is fluid management, demanding meticulous monitoring of fluid intake, current fluid status and urine output. This is vital for maintaining effective circulating volume, plasma osmolality within relatively narrow limits, and electrolytes within the physiological range.^10^ Effective fluid management needs to be appropriately tailored to the respective disease and its underlying pathophysiology.^11^ However, fluid overload, characterized by a positive cumulative fluid balance, is linked to increased mortality in critically ill patients and could be identified as an independent risk factor for acute kidney injury in the ICU.^12^

In the context of neurology, studies have demonstrated that acute brain dysfunction, encompassing delirium and coma, is associated with fluid overload.^13^ In various diseases commonly treated in a neurological ICU, such as traumatic brain injury and subarachnoid hemorrhage, a more positive fluid balances is associated with adverse outcomes.^14,15^

Nevertheless, current data on fluid management in stroke patients are limited despite indications that elevated brain water content during the early recovery phase may significantly contribute to enduring tissue damage. Even a Cochrane Review and guidelines acknowledge the absence of data to provide guidance on the volume and duration of parenteral fluid delivery.^16,17^ The recommendations are broad, emphasizing careful and multimodal monitoring of fluid balance, individualized replacement, and the maintenance of euvolemia.^18–20^ In a retrospective, single-center analysis focusing on patients with large hemispheric stroke and decompressive hemicraniectomy, the study revealed that cumulative net fluid balance independently contributed to a poor functional outcome at day 90.^21^

The management of fluid balance is therefore a crucial aspect of intensive care that significantly influences morbidity and mortality in critically ill patients. At present, there are no standardized guidelines for fluid management in stroke patients, specifically for those undergoing thrombectomy and subsequent ICU admission. This highlights the necessity for further research in this area to improve the quality of intensive care for critically ill patients with ischemic stroke and to optimize patient outcomes.

## Materials and methods

### Study design and setting

The study was conducted as a retrospective analysis within the framework of a single-center observational cohort study. The institutional and interdisciplinary Department of Intensive Care Medicine oversees 140 high-care ICU beds and treats approximately 5700 patients per year.

### Ethical approval and patient consent

The study protocol received approval from the local ethics committee (reference number WF-059/20) and adhered to the principles of the Declaration of Helsinki. Written informed consent was not required due to the retrospective study design, as all datasets were de-identified before processing and evaluation for the study.

### Participants and data sources

The ICU is equipped with Dräger^®^ monitoring systems (Infinity Delta, Lübeck, Germany). Demographic information, laboratory values, arterial blood gas samples, and vital parameters of patients are centrally stored in the Dräger^®^-supported Integrated Care Manager (ICM) software. Data access is facilitated through the use of the passively saved reporting database and the tool ICMiq from Dräger^®^. All stroke patients undergoing thrombectomy and subsequently admitted to the ICU within the study period (03/2015-06/2021) were included in the analysis. Functional and neurological outcomes were assessed at discharge from the hospital, encompassing the ICU, the stroke unit or the neurological normal ward, using the mRS and National Institutes of Health Stroke Scale (NIHSS), respectively. Functional outcome was again evaluated 90 days after stroke using mRS d90. Patients who lacked a documented NIHSS at first presentation or had an initial NIHSS of 42 (indicating arrival in a mechanically ventilated state), and those without NIHSS at discharge or mRS d90 were excluded. These exclusion criteria were applied to ensure the availability of valid NIHSS on admission and discharge, as well as mRS d90, for the assessment of favorable and unfavorable outcomes.

### Data analysis and statistics

Data processing was conducted in a standardized script-based manner utilizing R software environment (version 4.1.2). For the final composition of figures, Adobe Illustrator^®^ was used (Version 24.3). A detailed description of the data pre-processing steps was previously described.^22^

The recording of all fluid intake and urine output occurred at least once per shift and was carried out by the respective caregiver. For fluid intake, we considered all intentionally administered fluids within a 24-h period, ranging ideally from 6 AM–6 AM the following day. Urine output was measured for the same timeframe. In accordance with ICU protocols, a patient’s fluid balance was reset to zero upon admission to the ICU, irrespective of any fluids previously administered. Consequently, fluids administered by emergency medical services, in the emergency department, or during thrombectomy were excluded from the analysis. Any “partial” days on the day of admission or discharge were treated as “full” days. Daily fluid balance was subsequently calculated every day at 6 AM based on the data from the preceding 24 h. For patients with ICU stays exceeding 1 day, the mean daily fluid balance was calculated as the average of all daily fluid balance within the initial 5 days after ICU admission. This mean daily fluid balance value was used for further analysis.

Initial laboratory tests for all patients were conducted as early as possible, either upon arrival in the emergency department or upon admission to the ICU at the latest. Subsequent blood draws throughout the patient’s stay in the intensive care unit were also factored in, with the mean value of all tests calculated to facilitate inter-patient comparison.

The time to recanalization was defined as the duration between the patient’s admission to the hospital and the restoration of best blood flow in the previously occluded vessel.

An unfavorable functional outcome was defined as mRS d90 > 2, while an unfavorable neurological outcome was defined as NIHSS > 5 points at discharge. A multivariate linear regression model was trained to predict unfavorable outcomes with the maximal fluid retention per day within the first 5 days per patient. The model was adjusted for the following confounding variables: comorbid arterial hypertension and diabetes mellitus, sex, age, weight, NIHSS on admission, i.v. thrombolysis, TICI score, and mean serum creatinine levels.

We hypothesized that an average daily fluid balance exceeding 1 L is associated with worse neurological and functional outcomes. This decision was grounded on guidelines advocating for euvolemia maintenance, clinical considerations involving unmeasurable fluid losses (e.g. transpiration, fecal and exhaled air fluid), and statistical indications suggesting that 1 L represents the maximum tolerable daily fluid balance that is not associated with unfavorable outcomes.^18,23^

A multivariate logistic regression model was employed to assess unfavorable outcomes associated with a dichotomized average daily fluid balance of <1 L or >1 L, while controlling for the aforementioned confounding variables.

In assessing functional outcomes, the mRS d90 shift analysis was performed using an ANOVA, comparing patients with an average fluid balance of <1 L or >1 L during the initial 5 days in the ICU. The mRS d90 shift analysis was adjusted for sex, age, weight, NIHSS on admission, i.v. thrombolysis, TICI score, comorbid arterial hypertension and diabetes mellitus, and mean serum creatinine levels. To account for multiple comparisons, adjustments were made for False Discovery Rate. Categorical variables are reported as counts and percentages and were assessed for differences between patients with an average daily fluid balance of <1 L or >1 L using the χ^2^ test. Continuous variables are presented as mean and standard deviation (SD) and were compared between the aforementioned groups using the *t*-test after assessing for normal distribution. Data transformation, calculation, and visualization were performed in R (version 4.1.2 main packages: tidyverse, ggpubr, readxl, openxlsx, aov, gmodels, and lubridate). Significant results are indicated by *p < 0.05, **p < 0.01, ***p < 0.001, ****p < 0.0001.

### Data availability

Data are available from the corresponding author upon reasonable request.

## Results

### Recruitment and study cohort

During the study period 1252 patients diagnosed with acute ischemic stroke underwent endovascular thrombectomy. Subsequent to the procedure, 638 patients were admitted to ICU and among these 553 patients met the predefined inclusion criteria. Supplemental Figure 1 delineates the identification process of the study population, along with the applied inclusion and exclusion criteria. Patients’ demographics are shown in Table 1. The mean age was 72 years (SD ± 13.5 years), the gender distribution was approximately equal (54% female), and the average NIHSS on admission was 17.7 (SD ± 9.8). Fifty-seven percent of all patients received intravenous thrombolysis therapy. The average duration of ICU stay post-thrombectomy was 2.4 days (SD ± 2.2). The mean mRS and NIHSS at discharge was 4.2 (SD ± 2.1) and 9.4 (SD ± 7.8), respectively, and the mean mRS d90 was 4.1 (SD ± 1.9).

**Table 1.** Demographics of all included patients.

	All	Fluid balance < 1 L	Fluid balance > 1 L	*p*-Value
*N* (% female)	553 (54)	445 (52)	108 (62)	0.07
Age, years, mean (SD)	72.0 (13.5)	71.7 (13.8)	73.1 (12.4)	0.34
Weight, kg, mean (SD)	80 (17)	81 (18)	78 (16)	0.03
Stay in ICU, days, mean (SD)	2.4 (2.2)	2.1 (2.0)	3.4 (2.5)	<0.0001
Daily fluid intake, L, mean (SD; range)	1.1 (0.6; 0, 4.7)	1.0 (0.5; 0, 2.4)	1.7 (0.6; 0.7, 4.7)	<0.0001
Urine output, L, mean (SD; range)	0.6 (0.4; 0, 2.4)	0.7 (0.4; 0, 2.4	0.6 (0.3; 0, 1.9)	<0.001
Daily fluid balance, L, mean (SD; range)	0.6 (0.7; −1.7, 6.3)	0.3 (0.4; −1.67, 1.0)	1.7 (0.7; 1.0, 6.3)	<0.0001
NIHSS admission, mean (SD; range)	17.7 (9.8; 0, 42)	17.3 (9.7; 0, 42)	19.5 (10.1; 5, 42)	0.04
NIHSS discharge, mean (SD; range)	9.4 (7.8; 0, 42)	8.7 (7.3; 0, 42)	13.1 (9.3; 0, 42)	<0.001
NIHSS Δ, mean, (SD; range)	7.1 (11.0; −33, 42)	7.6 (10.9; −33, 42)	3.7 (11.1; −28, 42)	0.01
mRS at discharge, mean (SD; range)	4.2 (2.1; 0, 6)	4.0 (2.1; 0,6)	5.1 (1.7; 0, 6)	<0.0001
mRS d90, mean (SD; range)	4.1 (1.9; 0, 6)	3.9 (1.9; 0, 6)	4.9 (1.6; 0, 6)	<0.0001
i.v. thrombolysis, *N* (%)	316 (57)	253 (57)	63 (58)	0.86
Subtype, *N* (%)				
Large-artery atherosclerosis	241 (43.6)	204 (45.8)	37 (34.3)	0.03
Cardioembolism	255 (46.1)	201 (45.2)	54 (50)	0.39
Other determined aetiology	35 (6.3)	25 (5.6)	10 (9.3)	0.18
Undetermined aetiology	22 (4)	15 (3.4)	7 (6.5)	0.16
Side of infarction, *N* (%)				
Right	217 (39.2)	177 (39.8)	40 (37)	0.66
Left	221 (40)	178 (40)	43 (39.8)	1
Not applicable	115 (20.8)	90 (20.2)	25 (23.2)	0.51
Craniotomy, *N* (%)				
supratentorial	25 (4.7)	7 (2)	18 (10.1)	<0.0001
infratentorial	3 (0.56)	1 (0.3)	2 (1.1)	0.26
Thrombectomy, TICI, *N* (%)				
0	83 (15.0)	66 (14.8)	17 (15.7)	0.93
1	15 (2.7)	10 (2.2)	5 (4.6)	0.3
2a	53 (9.6)	38 (8.5)	15 (13.9)	0.13
2b	189 (34.2)	156 (35.1)	33 (30.6)	0.44
3	203 (36.7)	168 (37.8)	35 (32.4)	0.35
Not available	10 (1.8)	7 (1.6)	3 (2.8)	0.66
Passes, mean (SD; range)	2.2 (1.8; 0, 20)	2.1 (1.9; 0, 20)	2.4 (1.7; 0, 7)	0.08
Time to recanalization, min, mean (SD)	151 (144)	155 (150)	145 (132)	0.53
Preconditions, *N* (%)				
Hypertension	364 (66)	287 (64)	77 (71)	0.22
Diabetes mellitus	102 (18)	84 (19)	18 (17)	0.69
Hyperlipidemia	65 (12)	54 (12)	11 (10)	0.69
Atrial fibrillation	171 (31)	132 (30)	39 (36)	0.24
Stay on intensive care unit				
Ventilated patients, *N*, %	500 (90.4)	401 (90.1)	99 (91.7)	0.72
Duration of ventilation, h, mean (SD; range)	5 (6,4; 0, 37,7)	4.5 (6,2; 0, 37.7)	6.1 (6,6; 0; 27)	0.01
Horovitz-Index (PaO_2_/FiO_2_ ratio), mmHg, mean (SD, range)	358 (164; 76, 2191)	350 (133; 77, 893)	375 (212; 76, 2191)	0.16
Heart failure, *N* (%)	8 (1.5)	5 (1.4)	3 (1.7)	1.00
Infections, *N* (%)	184 (33.3)			
Pneumonia	135 (24.4)	95 (21.4)	40 (37)	<0.01
Bronchitis	5 (0.9)	3 (0.67)	2 (1.9)	0.25
Urinary tract infection	33 (6)	28 (6.3)	5 (4.6)	0.65
Endocarditis	2 (0.36)	2 (0.45)	0 (0)	1
Combination	9 (1.6)	7 (1.6)	2 (1.6)	0.69

FiO_2_: fractional inspired oxygen; ICU: intensive care unit; i.v.: intravenous; TICI: thrombolysis in cerebral infarction; mmHg: millimeter mercury; *N*: number; NIHSS: National Institutes of Health Stroke Scale; NIHSS Δ: NIHSS difference between admission and discharge; mRS (d90): modified Rankin Scale (at day 90); PaO_2_: arterial oxygen partial pressure; SD: standard deviation.

### Fluid overload on ICU is associated with worse functional and neurological outcomes

**Figure 1.**
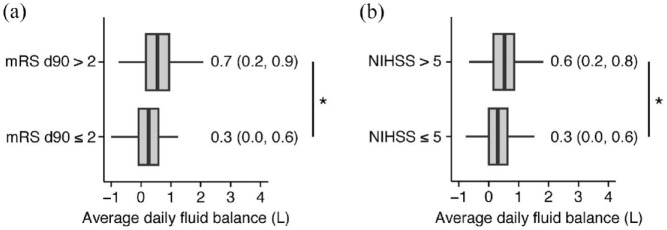
Average fluid retention within the first 5 days on ICU is associated with unfavorable functional and neurological outcomes: (a) patients were dichotomized into two groups based on mRS d90 scores: patients with mRS d90 > 2 and with mRS d90 ⩽ 2. The average fluid balance was employed to calculate an adjusted regression model for predicting mRS d90 and (b) patients were stratified based on their NIHSS at discharge, categorizing them into two groups: patients with NIHSS > 5 and patients with NIHSS ⩽ 5. Utilizing the average fluid balance, we calculated an adjusted regression model for predicting NIHSS. Median and range per group are displayed in the figure. For visual clarity, outliers have been omitted, but were fully included for statistical analyses. *p < 0.05.

Initially, a comparative assessment of fluid balance was conducted between patients exhibiting unfavorable and favorable functional outcomes (mRS d90 > 2 vs mRS d90 ⩽ 2) and neurological outcomes (NIHSS > 5 vs NIHSS ⩽ 5) after endovascular thrombectomy. Patients with unfavorable functional outcome had significantly higher average fluid balances than those with favorable functional outcome (Figure 1A: mRS d90 ⩽ 2: 0.3 ± 0.5 L, mRS d90 > 2: 0.7 ± 0.7 L, *p* = 0.02). Similar findings were observed for the NIHSS at discharge (Figure 1B: NIHSS ⩽ 5: 0.3 ± 0.5 L; NIHSS > 5: 0.6 ± 0.6 L; *p* = 0.03).

Subsequently, we explored the predictive capacity of average fluid balance on patient outcomes. Notably, fluid balance was significantly associated with adverse functional outcome (mRS d90 > 2). Specifically, for each 1 L increase in fluid balance, there was a 8% rise in the odds of patients experiencing mRS d90 > 2 (Supplemental Table 1; β-estimates = 1.08; 95% CI 1.04–1.11, *p* < 0.01). Additionally, we analyzed whether average fluid balance could also predict unfavorable neurological outcome at discharge (NIHSS > 5). Once more, the average fluid balance demonstrated a significant association with a NIHSS score > 5 at discharge (Supplemental Table 2; β-estimates = 1.12, 95% CI 1.05–1.18, *p* < 0.01).

### Average fluid excess >1 L during the first 5 days after thrombectomy is associated with poor functional and neurological outcomes

Patients were then dichotomized based on their fluid balance: those with an average daily fluid balance of more than 1 L and those with less than 1 L during the first 5 days on ICU (all descriptive parameters are shown in Table 1). Significant differences were observed between both groups for weight, but not for age and gender. The NIHSS at admission was significantly higher in the group with an average daily fluid balance > 1 L during the first 5 days on ICU (Table 1; fluid balance ⩽ 1 L: NIHSS 17.3 ± 9.7; fluid balance > 1 L: NIHSS 19.5 ± 10.1; *p* = 0.04). There were no significant differences regarding common preconditions, stroke characteristics, factors related to the mechanical thrombectomy or ICU adverse events except for different rates of large-artery atherosclerosis as the underlying etiology, incidence of pneumonia, and supratentorial craniotomy. Patients with an average daily fluid balance > 1 L exhibited a prolonged need for mechanical ventilation when necessary (fluid balance ⩽ 1 L: 4.5 ± 6.2 h; fluid balance > 1 L: 6.1 ± 6.6; *p* = 0.01). Patients with an average daily fluid balance > 1 L demonstrated a higher daily fluid intake compared to those with an average daily fluid balance ⩽ 1 L (Table 1; fluid balance ⩽ 1 L: fluid intake = 1.0 ± 0.5 L; fluid balance > 1 L: fluid intake = 1.7 ± 0.6 L; *p* < 0.0001). Urine output differ significantly between both groups (Table 1; fluid balance ⩽ 1 L: urine output = 0.7 ± 0.4 L; fluid balance > 1 L: fluid intake = 0.6 ± 0.3 L; *p* < 0.001).

**Figure 2.**
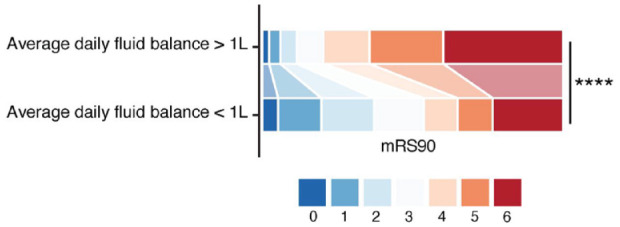
Shift analysis of mRS d90 between patients with euvolemic fluid balance and fluid overload. Each color represents a different mRS d90 score as indicated. In a multivariable ordinal regression analysis, a significant difference emerged between patients with an average fluid excess > 1 L and those with an average fluid balance ⩽ 1 L. The overall distribution of mRS scores favored a fluid balance ⩽ 1 L (AOR = 2.17; 95% CI 1.54–3.07; *p* < 0.0001), indicating an association between a lower fluid balance and improved functional outcomes at 90 days after thrombectomy

Our analysis revealed a shift in the distribution of mRS d90 scores, indicating improved functional outcomes in patients with an average daily fluid balance ⩽ 1 L compared to patients with an average daily fluid balance > 1 L (Figure 2 and Table 2; AOR = 2.17; 95% CI 1.54–3.07; *p* < 0.001). The shift in direction was consistent across all levels of mRS d90, revealing more patients with mRS d90 < 3 and less patients with mRS d90 ⩾ 4 when exhibiting an average daily fluid balance > 1 L. Consistently, patients with an average daily fluid balance > 1 L exhibited significantly higher odds of achieving a mRS d90 > 2 compared to those with an average daily fluid balance ⩽ 1 L (Table 3; AOR = 2.29; 95% CI 1.71–13.05, *p* < 0.01). Likewise, patients with an average fluid balance > 1 L had an increased odds for a NIHSS at discharge > 5 (Table 4, AOR = 1.16; 95% CI 1.05–1.29, *p* < 0.01) and a mRS at discharge > 2 (Supplemental Table 3, AOR = 1.15; 95% CI 1.08–1.23, *p* < 0.01).

**Table 2.** mRS d90 shift analysis.

	Odds ratio	*p*-Value	CI low	CI high
Daily fluid balance > 1 L	2.17	<0.01	1.54	3.07
Average serum creatinine levels	1.27	0.04	1.01	1.6
Age	1.04	<0.01	1.03	1.06
Sex	1.44	0.01	1.08	1.92
i.v. thrombolysis	0.7	0.01	0.53	0.92
Thrombectomy TICI 1	0.93	0.88	0.37	2.32
Thrombectomy TICI 2a	0.59	0.06	0.34	1.02
Thrombectomy TICI 2b	0.43	<0.01	0.28	0.65
Thrombectomy TICI 3	0.29	<0.01	0.19	0.43
NIHSS at admission	1.04	<0.01	1.02	1.05
Arterial hypertension	1.31	0.08	0.97	1.78
Diabetes mellitus	1.08	0.66	0.75	1.56

CI: confidence interval; i.v.: intravenous; mRS d90: modified Rankin Scale at day 90; NIHSS: National Institutes of Health Stroke Scale; TICI; thrombolysis in cerebral infarction.

**Table 3.** Logistic regression of unfavorable functional outcome (mRS d90 > 2).

	Odds ratio	*P*-value	CI low	CI high
Daily fluid balance > 1 L	2.29	<0.01	1.71	3.05
Average serum creatinine levels	1.31	0.01	1.05	1.65
Age	1.04	<0.01	1.03	1.05
Sex	1.32	0.06	1	1.77
Weight	1	0.48	0.99	1.01
i.v. thrombolysis	0.73	0.03	0.55	0.97
Thrombectomy TICI 1	1.06	0.9	0.4	2.84
Thrombectomy TICI 2a	0.57	0.05	0.33	0.99
Thrombectomy TICI 2b	0.45	<0.01	0.3	0.69
Thrombectomy TICI 3	0.34	<0.01	0.23	0.52
NIHSS at admission	1.03	<0.01	1.02	1.05
Arterial hypertension	1.06	0.69	0.78	1.45
Diabetes mellitus	1.25	0.21	0.88	1.79

CI: confidence interval; i.v.: intravenous; mRS d90: modified Rankin Scale at day 90; NIHSS: National Institutes of Health Stroke Scale; TICI; thrombolysis in cerebral infarction.

**Table 4.** Logistic regression of unfavorable neurological outcome (NIHSS > 5).

	Odds ratio	*P*-value	CI low	CI high
Daily fluid balance > 1 L	1.16	<0.01	1.05	1.29
Average serum creatinine levels	1.03	0.45	0.95	1.13
Age	1	0.11	1	1.01
Sex	1.08	0.14	0.98	1.19
Weight	1.0	0.24	1	1.01
i.v. thrombolysis	0.94	0.21	0.86	1.03
Thrombectomy TICI 1	0.96	0.85	0.66	1.4
Thrombectomy TICI 2a	0.88	0.24	0.73	1.08
Thrombectomy TICI 2b	0.86	0.06	0.73	1.01
Thrombectomy TICI 3	0.77	<0.01	0.66	0.9
NIHSS at admission	1	0.02	1	1.01
Arterial hypertension	1.01	0.84	0.91	1.12
Diabetes mellitus	1.11	0.1	0.98	1.01

CI: confidence interval; i.v.: intravenous; NIHSS: National Institutes of Health Stroke Scale; TICI: thrombolysis in cerebral infarction.

In light of potential biases arising from ICU length of stay, we categorized patients into two groups based on their duration: >72 h and <72 h. For patients with an ICU stay exceeding 72 h (*n* = 439), data from the initial 5 days were considered; for those with a shorter stay, data from all ICU days were included. Notably, patients with an ICU stay >72 h exhibited a mean daily urine output of 0.7 ± 0.4 L, whereas those with a stay <72 h showed a mean daily urine output of 0.5 ± 0.4 L (*p* < 0.05). Further analysis within the subgroup of patients with an ICU stay exceeding 72 h, dichotomized based on average daily fluid balance (< 1 L or > 1 L) during the initial 5 days, confirmed a significant deterioration in functional outcomes among patients with a daily fluid balance > 1 L, as evidenced by the mRS shift analysis (*p* < 0.0001).

### Laboratory parameters influence fluid balance following mechanical thrombectomy

A comparative assessment of laboratory parameters was conducted to identify factors potentially contributing to fluid retention in individuals undergoing endovascular thrombectomy. Building on previous findings, laboratory parameters were analyzed to discern differences between patients with an average daily fluid balance > 1 L and those with an average daily fluid balance ⩽ 1 L.

Whereas laboratory parameters indicating fluid overload, such as hemoglobin and hematocrit, showed no differences, indicators of systemic infection were significantly higher in the group with an average daily fluid balance > 1 L. C-reactive protein (CRP) values were elevated in both groups but patients with fluid retention showed significantly higher values (Supplemental Table 4; ⩽1 L, mean ± SD, 35.4 ± 34.7 mg/L; >1 L, mean ± SD, 60.7 ± 58.0 mg/L, *p* < 0.001). Arterial lactate was also significantly higher in patients with fluid excess but within physiological ranges for both groups (fluid balance ⩽ 1 L, mean ± SD, 1.1 ± 0.5; fluid balance > 1 L, mean ± SD, 1.3 ± 0.8, *p* < 0.001). In addition leukocyte counts fell within physiological ranges for both groups but were again found to be elevated in patients with fluid excess > 1 L (Supplemental Table 4; fluid balance ⩽ 1 L, mean ± SD, 10.300 ± 3300 cells/µL; fluid balance > 1 L, mean ± SD; 11.200 ± 3500 cells/µL, *p* = 0.037). However, patients with an average daily fluid balance > 1 L did not show elevated body temperature in comparison (fluid balance ⩽ 1 L, mean ± SD, 36.9°C ± 0.6°C; fluid balance > 1 L, mean ± SD, 36.8°C ± 0.6°C, *p* = 0.57).

To exclude the possibility that kidney failure was the primary factor driving fluid retention, we compared urea and creatinine between both groups, and found no significant differences in either parameter (urea: fluid balance 1⩽ L, mean ± SD, 17.5 ± 9.1; fluid balance > 1 L, mean ± SD, 19.5 ± 11.7, *p* = 0.1; creatinine: fluid balance ⩽ 1 L, mean ± SD, 1.0 ± 0.6; fluid balance > 1 L, mean ± SD, 1.0 ± 0.6, *p* = 0.74).

## Discussion

Presently, given the limited data on fluid management in stroke patients, stroke care guidelines advocate for maintaining homeostasis of physiological parameters. Furthermore, these guidelines do not offer specific recommendations for fluid management post-endovascular thrombectomy and in a neurointensive care setting.^16^

In this study, our objective was to examine the association between fluid balance post-endovascular thrombectomy and both functional and neurological outcomes in a neurological intensive care unit.

A cohort compromising 553 patients who met the predefined inclusion criteria was dichotomized based on their average daily fluid balance within the first 5 days after admission: either within the margin of 1 L or exceeding it. While patients’ preconditions, factors related to mechanical thrombectomy, and most stroke characteristics and adverse events in the ICU were comparable between these groups, stroke patients with a fluid balance exceeding 1 L experienced a significantly prolonged stay in the neurological intensive care unit. Additionally, they underwent supratentorial craniotomy more frequently, experienced a higher incidence of pneumonia, and required longer mechanical ventilation when necessary. Furthermore, these patients exhibited not only poorer functional and neurological assessments at discharge compared to those with an average daily fluid balance < 1 L but also an unfavorable functional outcome at day 90. So notably, in addition to established risk factors for unfavorable functional outcome at day 90, including age, sex, NIHSS at admission, and therapeutic interventions such as intravenous thrombolysis and successful endovascular thrombectomy, a daily fluid balance exceeding 1 L is linked to a shift toward a worse mRS d90 and NIHSS.

The identification of average daily fluid balance as a potential predictor of worse functional and neurological outcomes emphasizes the importance of post-interventional fluid management for stroke patients. This is particularly interesting as even a subtle yet statistically significant difference in average daily fluid balance could contribute to poor functional and neurological outcomes. These findings align with previous research conducted in a population with large vessel occlusion and hemispheric stroke.^21^

Numerous potential mechanisms may elucidate the association between excessive fluid management and unfavorable outcomes. Fluid accumulation can exacerbate cerebral edema, resulting in increased intracranial pressure and diminished cerebral perfusion.^24^ Furthermore, fluid accumulation has the potential to incite a systemic inflammatory response, resulting in endothelial dysfunction, disruption of blood-brain barrier, and neuronal damage in stroke patients.^25^ Fluid overload may impede cerebral autoregulation, rendering the brain more vulnerable to fluctuations in blood pressure and subsequent ischemic injury or hemorrhage.^26^

As the indications for mechanical thrombectomy expand, this evolution is anticipated to lead to an increasing number of endovascular treatments.^27^ In a meta-analysis, the effects of general anesthesia and conscious sedation during endovascular thrombectomy were compared, revealing a higher recanalization rate and superior outcomes in patients undergoing general anesthesia.^28^ Conversely, a recent pooled patient-level analysis of the EXTEND-Trials indicates inferior outcomes in patients undergoing general anesthesia during thrombectomy.^29^ While the best periinterventional anesthesiological approach remains unknown, the noted influence of fluid overload underscores post-procedural differences that warrant significant consideration, emphasizing the central role of fluid management in critically ill patients and its pivotal role in neurological intensive care.

Chronic kidney disease has emerged as one of the most pivotal modifiable risk factors for cardiovascular diseases and adverse long-term outcomes in stroke.^30,31^ Certain patient populations, such as black individuals and those with atrial fibrillation, experience worse long-term outcomes following a stroke.^32,33^ This highlights the crucial importance of meticulous fluid management in these patient populations. To address this confounding factor to a certain extent, we incorporated serum creatinine and serum urea levels into our model. We observed no differences in these parameters when comparing patients with an average fluid balance > 1 L to those with an average fluid balance of ⩽1 L. Recognizing the constraints of our kidney function data, one could hypothesize from these initial findings that increased fluid retention, irrespective of kidney function, may potentially forecast unfavorable neurological and functional outcomes.

In our study population, the subgroup with an average daily fluid balance > 1 L exhibited elevated levels of CRP, lactate, and leukocytes compared to patients with a more euvolemic fluid balance. These findings align with previous studies demonstrating that increased leukocyte counts and CRP levels on admission and short-term follow-up are linked to poorer outcomes and higher mortality in individuals undergoing stroke treatment with intravenous thrombolysis.^34,35^ Adverse outcomes and mortality are notably more frequent in stroke patients with elevated CRP levels before endovascular thrombectomy.^36^ In general, evidence indicates a higher incidence of lung or urinary tract infections in patients suffering from stroke, although the underlying mechanisms remain incompletely understood.^37^ This could be attributed to larger strokes volumes leading to more extensive neurological deficits and increasing the vulnerability of stroke patients to nosocomial infections.^38^ Furthermore, local inflammation in the central nervous system after stroke induced hypoxic cell death can cause a peripheral immune response, enhancing susceptibility to bacterial infections.^39–41^ In our study, the occurrence of pneumonia exhibited a statistical disparity, being more prevalent among patients with an average daily fluid balance exceeding 1 L. This could explain the fact of higher fluid administration as part of their intensive care management and the prolonged ventilation, or vice versa.^42^

However, in our population, the elevation of lactate and leukocytes in patients with fluid overload remained within normal ranges, and there were no discernible differences in body temperature. Additionally, patients with an average daily fluid balance < 1 L also showed increased CRP values (local laboratory normal threshold < 5 mg/L). An alternative explanation for an isolated elevation of CRP values in both subgroups could be attributed to the endovascular intervention, as it entails tissue damage from arterial vessel puncture and endovascular procedures, which may provoke an elevation of the acute phase protein CRP. While patients with an average daily fluid balance ⩾ 1 L exhibited a trend toward a higher number of passes during thrombectomy, there was no significant difference in time to recanalization. Further studies are needed to specifically explore the impact of early infections and isolated CRP elevation in the ICU on post-thrombectomy care for stroke patients.

### Limitations

The primary limitation of this study is its retrospective nature, which restricts our capacity to make conclusive assertions about the direct relationship between fluid overload and unfavorable outcomes. Consequently, the association between these two parameters may also be influenced by other factors, such as diuretic medication in patients with heart failure. Additionally, patients may have been admitted in a dehydrated state, leading to a shift in fluid intake and output, resulting in a more positive fluid balance. This is underscored by the observation that patients with an average fluid balance > 1 L had significantly higher fluid intake than those patients with an average fluid balance < 1 L, but simultaneously significantly lower urine output. Overall, the mean urine output was observed to be low in the entire patient population, possibly because uresis prior to ICU admission was discarded and therefore not accounted for. Center-specific fluid documentation which may overly prioritize partial days, especially with a mean ICU stay of only 2.4 ± 2.2 days, and overlook previously administered fluid, has the potential to introduce bias to interpretation. However, a sensitivity analysis conducted on patients with a length of stay exceeding 3 days, dichotomized based on average daily fluid balance, confirmed a shift toward unfavorable functional outcomes at day 90.

Another limitation of our study is its single-center design, which introduces the possibility of bias related to other procedural standards at our medical center and their impact on the selection of patients admitted to the ICU post-thrombectomy. For instance, patients’ pre-stroke condition, expressed as pre-stroke mRS, was not routinely collected and thus could not be incorporated into our analysis.

Additionally, our thrombectomy results lack the category TICI 2c due to its implementation in clinical routine after the initiation of patient recruitment. While the interventional success rates did not differ between the analyzed groups, this factor might still exert an influence on the extent of functional and neurological outcomes, constituting an unconsidered confounder.

Further limitations of the study involve potential biases related to the neurological deficits, which may affect adequate fluid intake in the study population. Consequently, patients with more severe strokes might face challenges in ingesting fluids independently and in adequate amounts, thereby increasing the risk of fluid imbalances and severe functional and neurological outcomes.

Moreover, CRP as a marker of systemic inflammation was significantly elevated in patients with higher fluid retention levels, potentially contributing to poorer outcomes. Prospective studies are warranted in the future to compare outcomes in patients undergoing liberal and restrictive fluid management.

## Conclusion

This study presents an association between an average daily fluid balance exceeding 1 L during the first 5 days of ICU stay and unfavorable functional and neurological outcomes in acute stroke patients undergoing endovascular thrombectomy. Fluid retention might serve as an additional predictive factor for both functional and neurological recovery. Overall, these findings emphasize the need for further research into post-interventional fluid management in acute stroke patients in the intensive care unit.

## Supplemental Material

sj-docx-1-eso-10.1177_23969873241271642 – Supplemental material for Fluid excess on intensive care unit after mechanical thrombectomy after acute ischemic stroke is associated with unfavorable neurological and functional outcomes: An observational cohort study Supplemental material, sj-docx-1-eso-10.1177_23969873241271642 for Fluid excess on intensive care unit after mechanical thrombectomy after acute ischemic stroke is associated with unfavorable neurological and functional outcomes: An observational cohort study by Maximilian Schell, Christina Mayer, Marcel Seungsu Woo, Hannes Leischner, Marlene Fischer, Jörn Grensemann, Stefan Kluge, Patrick Czorlich, Christian Gerloff, Jens Fiehler, Götz Thomalla, Fabian Flottmann and Nils Schweingruber in European Stroke Journal
