# Supplementary material for: Fluid excess on intensive care unit after mechanical thrombectomy after acute ischemic stroke is associated with unfavorable neurological and functional outcomes: An observational cohort study
Source: Eur Stroke J. 2024 Aug 16;10(1):74–83. doi: 10.1177/23969873241271642 (PMC11569545; doi:10.1177/23969873241271642)
Supplement: sj-docx-1-eso-10.1177_23969873241271642 – Supplemental material for Fluid excess on intensive care unit after mechanical thrombectomy after acute ischemic stroke is associated with unfavorable neurological and functional outcomes: An observational cohort study [file sj-docx-1-eso-10.1177_23969873241271642.docx]

**SUPPLEMENTARY DATA**

**Supplementary Figure 1. Study Population Flowchart: Identification, Inclusion, and Exclusion Criteria**

**
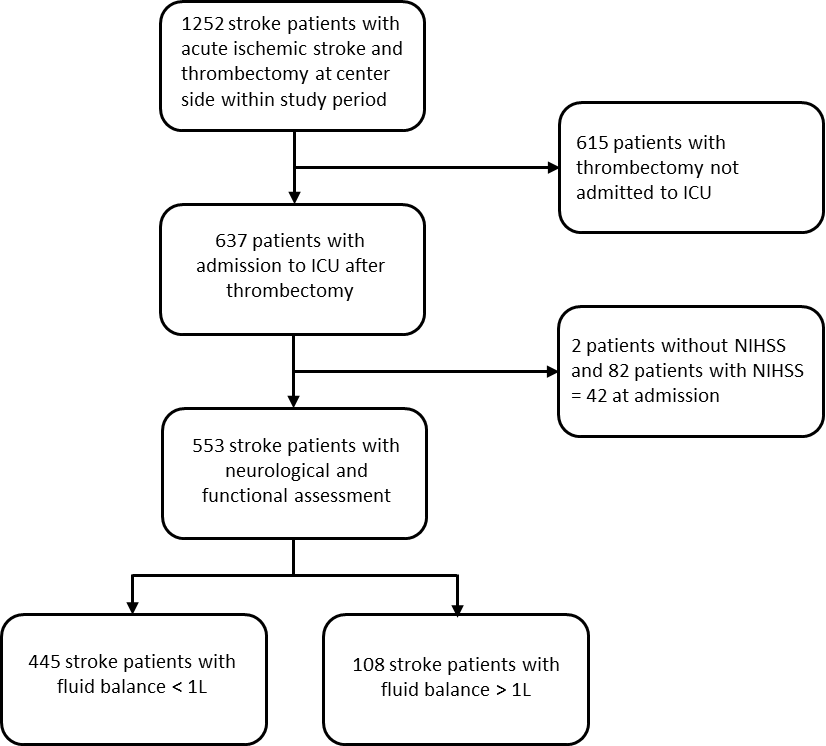
**

**Supplementary Table 1. Linear regression of unfavorable functional outcome (mRS d90 > 2).**

|  | β-estimates | *P*-value | CI low | CI high |
| --- | --- | --- | --- | --- |
| Daily fluid balance | 1.08 | **<0.01** | 1.04 | 1.11 |
| Average serum creatinine levels | 1.03 | 0.38 | 0.97 | 1.08 |
| Age | 1 | **<0.01** | 1.01 | 1.01 |
| Sex | 1.06 | 0.11 | 0.99 | 1.14 |
| Weight | 1 | 0.84 | 0.99 | 1.01 |
| i.v. thrombolysis | 0.93 | 0.36 | 0.87 | 1 |
| Thrombectomy TICI 1 | 1.01 | 0.47 | 0.86 | 1.38 |
| Thrombectomy TICI 2a | 0.93 | 0.29 | 0.81 | 1.06 |
| Thrombectomy TICI 2b | 0.88 | **0.02** | 0.8 | 1 |
| Thrombectomy TICI 3 | 0.83 | **<0.01** | 0.75 | 0.91 |
| NIHSS at admission | 1 | **<0.01** | 1 | 1.01 |
| Arterial hypertension | 1 | 0.84 | 0.92 | 1.07 |
| Diabetes mellitus | 1.05 | 0.3 | 0.96 | 1.14 |

CI = confidence interval, i.v. = intravenous, mRS d90 = modified Rankin Scale at day 90, NIHSS = National Institutes of Health Stroke Scale, TICI = Thrombolysis in Cerebral Infarction

**Supplementary Table 2. Linear regression of unfavorable neurological outcome at discharge (NIHSS > 5).**

|  | β-estimates | *P*-value | CI low | CI high |
| --- | --- | --- | --- | --- |
| Daily fluid balance | 1.12 | **<0.01** | 1.05 | 1.18 |
| Average serum creatinine levels | 1.04 | 0.39 | 0.95 | 1.13 |
| Age | 1 | 0.1 | 1 | 1.01 |
| Sex | 1.07 | 0.17 | 0.97 | 1.18 |
| Weight | 1 | 0.2 | 1 | 1.01 |
| i.v. thrombolysis | 0.95 | 0.26 | 0.86 | 1.04 |
| Thrombectomy TICI 1 | 0.99 | 0.95 | 0.68 | 1.44 |
| Thrombectomy TICI 2a | 0.9 | 0.3 | 0.74 | 1.1 |
| Thrombectomy TICI 2b | 0.86 | 0.07 | 0.74 | 1.01 |
| Thrombectomy TICI 3 | 0.78 | **<0.01** | 0.67 | 0.91 |
| NIHSS at admission | 1.01 | **0.02** | 1 | 1.01 |
| Arterial hypertension | 1.01 | 0.78 | 0.92 | 1.13 |
| Diabetes mellitus | 11 | 0.11 | 0.98 | 1.24 |

CI = confidence interval, i.v. = intravenous, NIHSS = National Institutes of Health Stroke Scale, TICI = Thrombolysis in Cerebral Infarction

**Supplementary Table 3. Logistic regression of unfavorable functional outcome at discharge (mRS > 2).**

|  | Odds ratio | *P*-value | CI low | CI high |
| --- | --- | --- | --- | --- |
| Daily fluid balance > 1L | 1.15 | **< 0.01** | 1.08 | 1.23 |
| Average serum creatinine levels | 1.01 | 0.6 | 0.96 | 1.07 |
| Age | 1 | **< 0.01** | 1 | 1.01 |
| Sex | 1.05 | 0.18 | 0.98 | 1.12 |
| Weight | 1 | 0.28 | 0.99 | 1.01 |
| i.v. thrombolysis | 0.93 | **0.04** | 0.88 | 1 |
| Thrombectomy TICI 1 | 0.99 | 0.9 | 0.78 | 1.24 |
| Thrombectomy TICI 2a | 0.98 | 0.69 | 0.86 | 1.11 |
| Thrombectomy TICI 2b | 0.9 | **0.04** | 0.82 | 0.99 |
| Thrombectomy TICI 3 | 0.8 | **< 0.01** | 0.76 | 0.92 |
| NIHSS at admission | 1.01 | **< 0.01** | 1 | 1.01 |
| Arterial hypertension | 1.02 | 0.63 | 0.95 | 1.1 |
| Diabetes mellitus | 1 | 0.28 | 0.99 | 1.01 |

CI = confidence interval, i.v. = intravenous, mRS = modified Rankin Scale, NIHSS = National Institutes of Health Stroke Scale, TICI = Thrombolysis in Cerebral Infarction

**Supplementary Table 4. Laboratory parameters of study population.**

| Parameters (mean (SD)) | Fluid balance < 1L | Fluid balance > 1L | *P*-value |
| --- | --- | --- | --- |
| Hemoglobin (g/dL) | 12,1 (1,8) | 12 (1,7) | 0.55 |
| Hematocrit (%) | 36,3 (5,1) | 36 (5) | 0.5 |
| CRP (mg/L) | 35.4 (34.7) | 60.7 (58.0) | **< 0.001** |
| Urea (mg/dL) | 17.5 (9.1) | 19.5 (11.7) | 0.1 |
| Creatinine (mg/dL) | 1.0 (0.6) | 1.0 (0.6) | 0.74 |
| Lactate (mmol/L) | 1.1 (0.5) | 1.3 (0.8) | **0.001** |
| Leukocytes (cells/µL) | 10.300 (3.300) | 11.200 (3.500) | **0.037** |
| Temperature (°C) | 36.9 (0.6) | 36.8 (0.6) | 0.57 |

CRP = C-reactive protein, SD = standard deviation
